# 보호출산제 시행과 젠더 및 보건의료 이슈

**DOI:** 10.4069/whn.2024.06.14

**Published:** 2024-06-28

**Authors:** Jiah Jeong

**Affiliations:** Department of Women-Gender Studies, Chungnam National University, Daejeon, Korea; 충남대학교 여성젠더학과

## 서론: 보호출산제 도입 배경

2023년 3월 23일, 헌법재판소는 “「가족관계의 등록 등에 관한 법률」 제46조 제2항 ‘혼인외 출생자의 신고는 모(母)가 해야 한다’는 위헌”이라며 낸 헌법소원사건(2021헌마975)에 재판관 전원일치 의견으로 헌법불합치 결정을 했다[1]. 남편이 있는 여성과의 사이에서 자녀를 낳은 A씨 등은 가족관계등록법에 따라 혼인 외 출생자들의 출생신고를 할 수 없게 되자 2021년 8월 자녀들과 함께 헌법소원을 내었고, 이에 헌법재판소는 “혼인 중 여자와 남편 아닌 남자 사이에서 태어난 자녀는 출생신고가 곤란한 상황이 발생해 사회보험•사회보장 수급을 제대로 받지 못하고 주민등록이나 신분 확인이 필요한 거래를 하기 어려우며 학대당하거나 유기되기 쉽고 범죄의 표적이 될 가능성이 높다”면서 “혼인 외 출생자인 청구인들의 태어난 즉시 ‘출생 등록될 권리’를 침해한다”고 설명했다. 또 “태어난 즉시 ‘출생 등록될 권리’는 ‘출생 후 아동이 보호받을 수 있을 최대한 이른 시점’에 아동의 출생과 관련한 기본 정보를 국가가 관리할 수 있도록 등록할 권리로서 아동이 사람으로서 인격을 자유로이 발현하고, 부모와 가족 등의 보호 하에 건강한 성장과 발달을 할 수 있도록 최소한의 보호장치를 마련하도록 요구할 수 있는 권리”라고 강조했다.

또, 지속적으로 우리 사회에 충격을 주었던 영아살해ˑ영아유기죄가 오랜 기간 논의되다가 이를 폐지하는 내용의 ‘형법’ 개정안이 2023년 7월 18일 국회 본회의를 통과했고 2024년 2월 9일부로 폐지되었다. 기존의 ‘형법’에서 영아살해죄는 ‘직계존속이 치욕을 은폐하기 위하거나 양육할 수 없음을 예상하거나 특히 참작할 만한 동기’로 영아를 살해 또는 유기한 경우, 최대 징역 10년으로 일반 살인죄나 유기죄보다 감경해 처벌하고 있었는데, 최근 영아가 태어나고도 출생신고 없이 유기되거나 살해되는 사회적 문제에 대한 높은 국민적 관심과 우려를 반영해 법 조항을 개정하여 영아를 살해•유기한 경우에도 일반살인•유기죄를 적용하도록 함으로써 저항 능력이 없거나 현저히 부족한 사회적 약자인 영아의 생명권을 두텁게 보호하도록 했다[2].

이러한 사회적 요구와 법제 개편은 부모에게만 출생신고 의무가 주어졌던 것에 더하여 「가족관계의 등록 등에 관한 법률」을 개정하여, 출산을 맡았던 의료기관의 장에게도 신고 의무가 주어지도록 당위성을 부여해 주었다. 또 같은 해 경기도 수원의 한 아파트 냉장고에서 영아 시신 2구가 발견된 사건과 같이 지속적으로 발생하는 영아유기ˑ살해사건, 미신고 아동 실태를 감사원에 제보하고 보건복지부 감사를 이끌어낸 ‘사회적 부모’ 프로젝트 활동, ‘2015년부터 2022년까지 8년간 미신고 아동이 총 2,236명’이라는 보건복지부 감사 결과가 사회에 보고되면서 국회를 표류하던 출생통보제는 2023년 6월 30일 국회의원 재석 267명에 찬성 266표, 기권 1표의 압도적인 찬성으로 통과됐다.

더불어 출생통보제가 도입되면 출산 사실을 알리고 싶지 않은 임부가 의료기관이 아닌 병원 밖 출산으로 위기임산부가 증가할 우려가 제기되어 보호출산제도가 도입되어야 한다는 필요성이 급물살을 탔고, 이는 헌법소원사건(2021헌마975)에서 재판관 소수의견에서도 찾아볼 수 있다. 또한 정부에서도 국민적 여론집중에 따라 의료기관 출생통보제의 준비 계획 및 실행 방안에 대해 논의하면서 경제적•사회적•심리적 이유 등으로 아이를 키우기 어려운 상황에 놓인 임산부가 불가피한 경우 자신을 밝히지 않고 의료기관에서 출산할 수 있도록 하여 산모와 아동의 생명과 건강을 보호하는 것을 목적으로 하는 보호출산제 도입을 검토하였다. 이는 의료기관 출생통보제 시행으로 초래될 수 있는 병원 밖 출산 증가 등을 방지하고 빈틈없이 아동을 보호하기 위한 법률로, 위기임산부 상담•지원과 보호출산 지원을 담고 있다[3]. 이러한 입법론이 반영된 결과 지난 2023년 10월 6일 국회 본회의에서 재석 230명에 찬성 133표, 기권 64표로 ｢위기 임신 및 보호출산 지원과 아동 보호에 관한 특별법｣(약칭: 위기임신보호출산법) 제정안이 의결되었고, 출생통보제와 보호출산제는 2024년 7월 19일부터 시행된다.

## 출생통보제와 보호출산제 주요 내용[4,5]

통계청 인구동향조사 결과 2021년 기준 출생아의 99.8%가 의료기관에서 출생했고, 이러한 현상을 고려하여 출생통보제가 마련되었다. 의료기관장은 출생일로부터 14일 이내에 건강보험심사평가원(이하 “심평원”)에 출생 정보를 통보해야 하고, 통보한 출생 아동 정보를 바탕으로 시‧읍‧면장은 부모의 출생신고 여부를 확인하여 누락 시 최고하고, 필요시 직권 출생 기록한다. 주요 내용은 다음과 같다.

○ (통보주체‧기한‧대상) ①의료기관의 장은 출생 14일 이내 심평원에 통보, ②심평원은 시‧읍‧면장에게 통보

○ (통보내용) 母의 성명․주민번호, 출생아의 성별‧수(數)‧출생연월일시 등

○ (통보방법) ①(의료기관→심평원) 심평원에서 운영하는 전산정보시스템 이용,

② (심평원→시•읍•면장) 행정정보 공동이용센터를 통해 시‧읍‧면장에 통보

○ (최고) 출생정보를 통보받은 시‧읍‧면장은 출생 신고기간(1개월) 내 신고되지 않을 경우, 신고의무자에게 최고 통지

○ (직권출생기록) ①최고기간(7일) 내 未신고, ②신고의무자 특정이 불가능한 경우 등, 시‧읍‧면장이 감독법원의 출생확인을 받은 후 직권으로 출생기록

보호출산제는 병원 밖 출산 및 아동유기를 방지해 여성과 아동의 생명•건강을 보호하기 위해 마련되었고, 제1장 제1조(목적)에서 적시된 바와 같이 경제적ˑ심리적ˑ신체적 사유 등으로 인하여 출산 및 양육에 어려움을 겪고 있는 임산부의 안전한 출산을 지원하고 그 태아 및 자녀인 아동의 안전한 양육환경을 보장함을 목적으로 제정되었다. 위기임산부에 대한 상담, 보호출산, 아동보호, 출생증서의 정보공개, 그리고 이의 실현을 위한 국가와 지방자치단체의 책무를 규정하였고, 주요 내용은 다음과 같다.

○ (상담) 위기임산부가 신중하게 보호출산을 선택할 수 있도록 충분한 상담 및 임신‧출산‧양육지원 정보 제공을 위한 상담체계 구축‧운영

① 위기임산부를 위한 지역상담기관이 지정되고, 위기임산부는 언제든지 지역상담기관에서 출산 후 직접 아이를 키울 수 있도록 돕는 상담, 정보 제공 및 서비스 연계를 받을 수 있다. 각종 법령에 따른 사회보장 급여와 직업•주거를 위한 지원, 의료비 지원 같은 경제적 지원뿐 아니라 양육비 이행 확보를 위한 지원 같은 법률적 지원까지 상담과 서비스 연계를 받을 수 있도록 규정하였다.

② 지역상담기관을 지원하는 중앙상담지원기관도 설치된다. 중앙상담지원기관에서는 위기임산부의 출산•양육 지원과 아동 보호를 위한 상담 내용, 절차를 개발•보급하고 교육을 실시한다.

③ 지역상담기관에서는 위기임산부가 출산 전후에 주거와 돌봄을 지원받을 수 있도록 한부모가족 복지시설이나 사회복지시설에 연계할 수 있게 된다. 이에 더해 관계 법령에 따라 출산 후 산후조리도 지원할 수 있다.

○ (보호출산) 보호출산을 신청한 산모가 의료기관에서 가명으로 산전검진 및 출산할 수 있도록 비식별화 조치, 비용 지원 등 실시

① 임산부가 보호출산을 원하는 경우에는 지역상담기관에서 보호출산 절차와 법적 효력, 자녀의 알 권리와 그것이 자녀의 발달에 미치는 영향 등 자녀의 권리 등에 대해 다시 상담을 한 뒤 보호출산 신청을 받는다.

② 보호출산을 신청하면 가명과 관리번호(주민등록번호를 대체할 수 있는 가명 처리를 위한 번호)가 생성되고, 임산부는 이 가명과 관리번호를 사용해 의료기관에서 산전 검진과 출산을 할 수 있다. 이 경우 의료비는 전액 지원된다.

○ (아동보호) 태어난 아동에 대한 지자체 인도, 출생등록 및 보호조치(입양, 시설보호 등)의 절차 마련

① 아이가 보호출산으로 태어난 후 임산부는 최소한 7일은 아동을 직접 양육하기 위한 숙려기간을 가져야 하고, 이 기간이 지난 후에 지방자치단체에 아동을 인도할 수 있다. 아동을 인도받은 지방자치단체장은 지체 없이 「아동복지법」에 따른 보호조치를 하여야 하며, 입양 등의 보호 절차를 밟게 된다.

② 보호출산을 신청했던 어머니는 아동이 「입양특례법」상 입양 허가를 받기 전까지 보호출산을 철회할 수 있다.

○ (기록관리) 상담‧출생 기록 작성‧보관, 출생증서 공개 절차‧요건 등

① 어머니는 보호출산을 신청할 때 자신의 이름, 보호출산을 선택하기까지의 상황 등을 작성하여 남겨야 한다. 이때 작성한 서류는 아동권리보장원에 영구 보존되며 보호출산을 통해 태어난 사람은 성인이 된 후에, 또는 법정대리인 동의를 받아 이 서류의 공개를 요청할 수 있다.

② 이때 생모가 동의하면 서류 전체가 공개되고, 동의하지 않거나 생모의 동의 여부를 확인할 수 없는 경우에는 인적 사항을 제외하고 공개된다. 다만, 사망 등으로 생모의 동의 여부를 확인할 수 없으며 의료상 목적 등 특별한 사유가 있을 때에는 전체를 공개할 수 있다.

## 보호출산제와 젠더 이슈

많은 나라들이 위기임신에 대한 사회적 안전망의 필요성을 절감하고 국가적 노력을 기울이고 있다. 독일, 프랑스, 미국 등 주요국에서도 다양한 방식으로 ‘익명 출산에 대한 권리’를 보장하고 있는데, 특히 독일에서는 2013년 신뢰출산법(Vertrauliche Geburt)을 제정해 2014년부터 시행하면서 위기임산부 보호를 법으로 정하고 있고, 위기임산부 지원을 위한 ‘임신갈등 상담소(Pro Familia Berlin)’를 설치, 운영하고 있다. 친모가 익명으로 출산 시 정부가 아이의 성을 정하고, 자녀가 16세가 되면 친생모 신상정보가 포함된 출생증명서 열람이 가능하도록 해 아동의 부모 알 권리를 보장한다. 또한 임신갈등 상담소는 홈페이지와 24시간 전국 핫라인 등을 통한 익명 상담으로 신뢰출산제 및 지원 정책에 대한 정보를 충분히 제공하여 임신 중단을 포함한 임산부의 자기결정을 최대한 지원하고, 양육이 불가능할 경우에만 입양이나 신뢰출산제를 통해 해결되도록 하고 있다. 또, 자녀 출산 이후 거주지를 기준으로 주치의를 지정할 수 있으며, 크고 작은 질병 발생 시 주치의를 통해 치료를 받을 뿐 아니라 성장검사를 받을 수 있는 ‘아동 주치의 제도’가 마련되어 있고, 대부분의 아동 의료비를 지원해 주고 있다. 프랑스의 익명 출산제(L’accouchement sous X)와 미국의 영아 피난소(Safe Haven) 법도 이와 유사하나, 익명성은 보호하지만 친생모의 정보도 익명 처리하는 것이 다른 점이다[6-10].

이렇듯 위기임산부 보호라는 사회적 요구는 전 세계 공통적으로 논의되는 사안인 만큼 우리나라 정부도 법제 정비와 더불어 ‘출생통보 및 보호출산 제도 시행 추진단’을 결성해 출생통보제와 보호출산제 시행을 위해 정부ˑ지자체ˑ법원 행정처가 힘을 모으고, 유관기관(국민건강보험공단, 심평원, 사회보장정보원, 아동권리보장원)과의 협업으로 2024년 7월 19일 예정된 제도 시행을 차질없이 준비 중이라고 밝혔다[5].

그러나 보호출산제 시행을 앞두고 우리 사회에는 여러 문제가 제기되고 있다. 보호출산제는 장애아동에 대한 보호조치, 이주여성과 아동의 공백 해결을 위한 입법이 필요하다는 문제 제기와 함께 ‘여성도 아동도 보호하지 못하는 제도’라는 견해가 대표적이다[11-14]. 특히 여성과 아동의 권리에 대한 비판은 「위기임신보호출산법」 제정 전후 시민사회단체와 여성변호사회[13], 국회[14] 등 각종 포럼 등에서 공통적으로 확인할 수 있는데, 이 법은 근본적으로 미혼모를 숨어서 출산하게 하고 양육 포기의 수많은 사유들을 해결하는 데는 눈 감고 있어 근본적 해결이 되지 않을뿐더러, 보호출산으로 태어난 사람이 출생증서 공개를 청구하여도 생모와 생부의 동의를 받아야 공개 가능하여 아동의 정체성을 알 권리를 심각하게 침해하고 있다는 것이다. 실제로 2014년부터 익명 출산이 가능한 독일의 경우 우리 나라의 보호출산제와는 달리 익명 출산과 아동의 부모 알 권리를 균형 있게 보장하고 있으며, 신뢰출산을 최후의 수단으로 권고하고 있어 제도의 부작용을 최소화할 수 있는 방안을 마련했다고 볼 수 있다. 여기에는 우리가 중요하게 다루어야 하는 사회적 의제인 ‘정상가족 담론’에 대한 논의가 필요하다[12].

독일에서는 혼인 외의 자와 혼인 중의 자에 대한 법체계 내에서의 구별을 완전히 없앴고, 그로써 차별과 편견이 사라지게 하는 파생적 효과를 가져오고 있다. 그러나 우리나라의 보호출산제는 근본적으로 보호출산에서 보호하고자 하는 위기임산부를 혼인 외 비정상 가족으로 상정하고, 미혼모를 ‘숨어서 출산하고 싶어 하고 양육을 포기하는 사람’으로 전제하고 있다. 미혼모는 철없고 부도덕하며 자기 관리가 안되는 것으로 폄하되고, 정조를 중시하는 유교 문화가 뿌리 깊은 우리 나라에서는 사회의 지탄의 대상이 되면서 ‘정상가족 이데올로기’와 연결되어 차별과 편견의 대상이 되어왔다. 이는 「건강가정기본법」에서 적시된 바와 같이 ‘정상가정’을 기본값으로 상정하여 전반적인 법 제도가 설계되어 있어 법률혼만을 법에서 인정하는 혼인으로 보고 그로부터 파생된 가정만을 법에서 인정하여 보호하고 있기 때문에, 결국 소위 ‘정상가정’의 개념 아래 혼인외 자와 혼인 중 자의 차별을 전제로 한 법일 수밖에 없다. 따라서 양육 포기의 수많은 사유에 대한 어떠한 보장적 장치들이 마련되어 있지 않고 법 제정 과정에서도 논의되지 않았다는 점이 문제적이라 생각한다.

또한, 출산통보제 시행으로 의료기관 밖 출산의 위험에 노출될 가능성이 있는 위기임산부를 위해 제정된 만큼, 「위기임신보호출산법」 내 여성의 당사자성과 주체성을 실질적으로 보호할 수 있는 장치들을 보완하여야 할 것이다. 임신부터 출산에 이르기까지 상담을 통해 신체적ˑ경제적ˑ심리적 지원을 할 수 있다고 하나, 임신 중지 및 재생산에 대한 여성의 결정권을 실질적으로 보장하는 장치들이 마련되어 있지 않다. 2019년 4월 11일 헌법재판소의 낙태죄 헌법불합치 결정 이후 당해 연도까지 대체입법이 마련되지 않아 2021년 1월 1일부터 낙태죄는 효력을 상실하였다. 국가 책무로서 대체입법이 마련되지 않은 현 상황에서 의료 현장에서는 안전한 임신 중단을 위한 장치가 부재하고, 유산 유도제 등 불법 약물이 온라인을 통해 유통되는 등 위기임산부를 더욱 양산하고 있는 상황이다. 실제로 Kim [15]은 최근 5년 내 임신 중단을 경험한 만 19–44세 여성 602명을 대상으로 한 연구에서, 가장 최근 임신 중단 시 연령은 20대 이하가 52.7% (19세 이하 4.5%p 포함), 혼인 상태는 비혼이 51.3%, 주관적으로 인지하는 사회계층은 상층이 6.6%, 중층이 42.7%, 하층이 50.7%라고 보고하여 보호출산제가 보호하고자 하는 대다수의 위기임산부가 포함되어 있음을 알 수 있다. 더 이상 사회적 합의가 이루어지지 않는다는 이유로 여성의 임신을 중지할 권리에 대한 논의를 미루어 더 많은 위기임산부를 양산해서는 안 된다.

마지막으로, 「위기임신보호출산법」에도 여전히 아버지의 존재는 철저히 숨겨져 있고, 출산 후 돌봄의 책임은 오롯이 출산한 여성에게만 주어지고 있음을 알 수 있다. 물론 위기임산부가 아동을 직접 양육할 수 있도록 「국민기초생활보장법」, 「한부모가족지원법」, 「국민건강보험법」, 「모자보건법」 등에서 일정 수준 지원받을 수 있으나, 이는 보편적 지원으로 위기임산부의 특별 상황을 고려한 전문적 지원이라 할 수 없고, 출산 여성에게만 돌봄을 책임지게 하고, 책임지지 않는 여성을 비난하는 사회 문제에서 벗어날 수 없다. 또 제7조 2항에서 「양육비 이행확보 및 지원에 관한 법률」에 따라 양육비 이행 확보를 지원한다고 적시되어 있으나, 이 또한 양육비 이행이 실효적이지 않음이 자명하다. 여성가족부에서 발표한 2021년 한부모가족 실태조사[16]에 따르면 법적 양육비를 지급해야 함에도 받은 적이 없다고 응답한 비율이 72.1%로 상당수 차지하고 있는바, 현실에서도 실효적이지 않은 법 조항을 적시하는 수준이다. 양육비는 헌법에서 보장되는 생명권과 행복추구권과 직결되는 문제이지만 현재의 법과 제도에서는 양육비를 주지 않아도 그다지 문제가 되지 않는 인식이 강하게 자리 잡고 있는 것도 우리 사회가 풀어내야 할 과제이다. 출산에 있어 아버지의 책임이 부재한 상태를 더 이상 방치해서는 안 될 것이다.

## 보호출산제와 보건의료 이슈

보건의료 분야 종사자는 새롭게 시행되는 출생통보제와 보호출산제 추진 배경과 절차를 정확히 이해하고, 준비해야 한다. 보호출산을 신청한 위기임산부에게 전산 관리번호와 가명을 부여하여 출산 사실이 기록되지 않도록 하고, 위기임산부에게 ‘임산부 확인서’를 발급하여 의료기관에서 가명으로 진료받을 수 있도록 하는 등 의료 현장에서의 시행 절차와 태도에 대한 충분한 교육 또한 필요하다. 또한, 법에서 규율하고 있는 대상자들은 우리 사회가 보호해야 하는 사회적 약자로, 한부모와 미혼모에 대한 편견과 차별을 없애고 다양성을 수용하는 태도가 필요하다. 특히, 일차의료기관의 의료인은 위기임산부를 최초 또는 초기부터 대면하는 접점으로서 임산부가 자기결정권과 모성권을 부정당하거나 존중받지 못한다고 느끼지 않도록 임산부 개개인의 삶에 대한 인정과 존중, 편견 없는 태도를 가질 것이 강하게 요구된다. 의료법은 의료인에게 생명윤리나 의료에서 윤리적 문제들에 대한 인식을 갖추기 위해 초점을 맞추고 있으나, 민감한 사회적 감수성에 기초한 공감과 이해, 편견 없는 사고를 기르는 것에 역량을 기울일 필요가 있다.

더불어 독일에서 시행하고 있는 ‘아동 주치의 제도’와 서울시에서 2018년부터 시행 중인 ‘서울 아기 건강 첫걸음 양육지원 사업’[17]을 벤치마킹한다면 위기임산부 지원에 의료계의 역할이 더 강화될 것이다. ‘서울아기 건강 첫걸음 양육지원 사업’은 출산한 모든 임산부와 신생아를 대상으로 4주 이내에 전문 간호사가 가정방문을 실시하고(보편방문), 임산부와 신생아에게 건강 위험요인이 있으면 신생아가 2세가 될 때까지 25회(지속방문) 방문을 통하여 건강관리를 하는 사업이다. 이 사업은 임산부가 산전과 후에 겪는 사회적, 심리적 어려움에 대처하고 신생아 양육역량을 강화할 수 있도록 보편방문, 지속방문, 부모 모임, 연계 서비스 등을 제공하며, 영유아가 최선의 건강 발달을 할 수 있도록 다양한 교육을 제공한다.

또 의료기관 접근권도 중요한 이슈이다. 국가에서는 임산부에게 건강한 태아의 분만과 산모의 건강 관리를 위하여, 산전ˑ후 관리를 위한 진료비 일부를 국민행복카드로 임신 1회당 100만 원(다태아 임산부는 140만 원 지원)을 지원한다. 그러나 양육 미혼모 146명 중 41명은 국민행복카드를 모르거나 사용하지 않는다는 한국미혼모가족협회 조사 결과[14]는 임산부에게 정확한 의료정보를 제공하는 사회적 안전망이 필요함을 보여준다. 한편 2021년 국민건강영양조사에 따르면 최근 1년 동안 본인이 병의원(치과 제외) 진료(검사 또는 치료)가 필요하였으나 받지 못한 분율(%)인 미충족 의료율(병의원)이 6.7%라고 보고되었다[18]. 미충족 의료의 원인은 의료기관 접근성, 의료비 부담 등 물리적, 경제적 요인뿐 아니라 환자의 지식, 태도, 불안이나 우울 등의 정서적 요인, 진료 또는 2차 피해에 대한 두려움, 사회적 지지 결여 등으로 의료기관을 찾지 못하는 수용성 등 다양한 요소들과 관련이 있다. 미충족 의료율에 위기임산부가 차지하는 비율에 대한 조사 결과는 없으나, 실제로 한국미혼모가족협회 조사에서 양육 미혼모들이 병원비가 부담스럽거나, 병원을 가기가 꺼려지거나, 병원을 가는 것 자체가 두려워서 병원 진료를 받지 않는다고 답하고 있는 것으로 미루어[14] 미충족 의료율이 높을 것으로 추정된다. 즉 위기임산부와 같이 미충족 의료 경험이 높은 취약계층에 대한 경제적 지원을 뛰어넘어 다양한 차원의 분석과 해결책, 그리고 그를 뒷받침할 촘촘한 사회안전망이 필요하다는 것을 시사한다.

## 결론: 보호출산제 전망과 제안

보호출산제는 그동안 위기 상황에 놓인 임산부들의 출산ˑ양육을 돕고, 긴급한 상황에 대해 민간 현장 활동단체에서 담당하던 사회적 안전망을 국가가 체계적으로 보호하고자 했다는 데 의의가 있다. 특히 사회적 취약계층인 미혼모, 청소년 미혼모와 같이 임신과 출산에 대한 불안정, 차별 및 낙인을 겪는 경우 원 가족뿐 아니라 사회적 지원체계가 불비한 우리 사회에서 어떠한 보호막 없이 개인적으로 더욱 고립되고 취약해질 수밖에 없는데, 국가가 개입하여 그들을 보호하고 조력할 수 있는 시스템이 마련되었다는 것은 다행스러운 일이다.

정부 발표에 따르면 미혼모 상담 등 관련 업무를 3년 이상 수행하여 전문성을 보유한 비영리법인 또는 사회복지법인이 위기임산부 (지역)상담기관으로 지정을 받을 수 있고, 위기임산부 직통전화(핫라인)를 운영하여 도움이 필요한 위기임산부가 야간에 연락하더라도 상담기관이 언제든지 대응할 수 있게 하는 등의 내용이 보강된 「위기임신보호출산법 시행령 및 시행규칙」 제정안과 6개의 하위 법령 일부 개정에 관한 보건복지부령안을 입법 예고했다[5]. 이제 우리는 보호출산제와 관련된 여러 우려와 목소리에 귀 기울여 법 제정의 근본 목적인 위기임산부와 태아 및 자녀를 실효적으로 보호할 수 있는 방향으로 정책을 수립하고 제도가 정착되도록 사회안전망을 더욱 견고히 만들어야 한다. 제정된 법안들이 탁상공론이 되지 않도록, 여성과 아동의 권리 침해 등 제기되는 많은 우려 점을 점검하면서 계속 보완할 필요가 있다. 보호출산제에 대해 각계에서 우려가 많은 만큼 시행 초기부터 민감성을 가지고 현장의 목소리에 귀 기울이고 보완해 나가야 할 것이다.

보호출산제는 보호출산을 우선적으로 선택할 수밖에 없는 구조를 가진 법으로 보인다는 비판도 있다. 그러나 의료기관 밖 출산의 위험에서 벗어나 보호출산을 최후의 수단으로 선택하는 위기임산부에게는 의료기관에서의 안전한 출산을 보장해 주는 보호장치인 동시에, 임신과 출산뿐 아니라 양육과정에서 발생할 수 있는 보육 및 경제적 어려움 등에 대해 사회복지 서비스를 제공받을 수 있는 최소한의 법적 조치이다. 그러나 위기임산부의 경우 사회에 드러나기를 극도로 꺼리기 때문에 병의원에 가지 않는 등 각종 정부 임신 지원 정책을 제공받지 못하는 문제가 있으므로, 위기임산부에 대한 체계적인 등록ˑ관리를 통해 그들이 겪는 금전적 문제, 심리 지원, 양육방법에 대한 교육, 주거 문제, 교육비, 돌봄 서비스 확대, 건강검진 서비스 등 다양한 사정과 복잡한 문제들로부터 양육의 어려움을 더는 실질적 지원이 수반되어야 하겠다.

더불어 보호출산제 시행을 앞둔 지금, 위기임산부에 대한 국가적 책무와 사회적 안전망이 강화되어 여성에게만 출산에 대한 책임을 전가하지 않고, 정상가족 이데올로기, 안전하게 임신을 중단할 권리, 돌봄에서 모성과 부성의 역할을 끊임없이 논의하면서 동시에 현실을 반영하는 법 개정이 수반되기를 기대해 본다.
